# Supplementary figures and images for: Comparative Transcriptomics and Gene Knockout Reveal Virulence Factors of Arthrinium phaeospermum in Bambusa pervariabilis × Dendrocalamopsis grandis
Source: J Fungi (Basel). 2021 Nov 24;7(12):1001. doi: 10.3390/jof7121001 (PMC8705590; doi:10.3390/jof7121001)

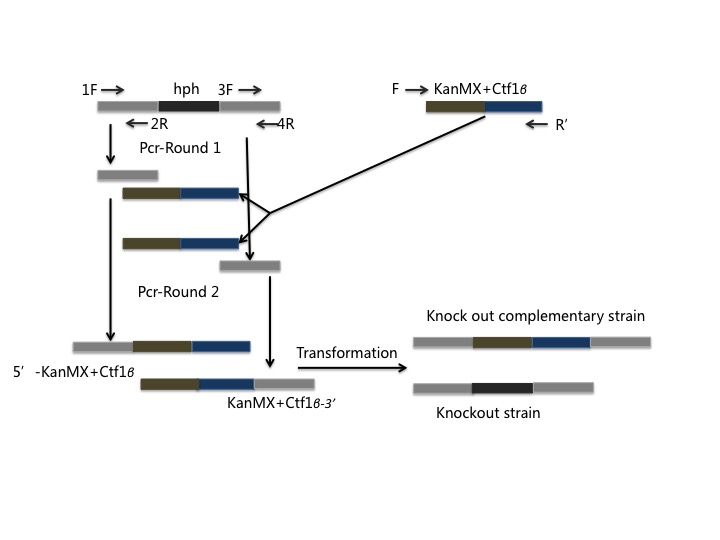

Supplement: Supplementary file 1 [file jof-07-01001-s001.zip › Figure S1 Illustration of the construction process of knockout out complementary vector.jpg]

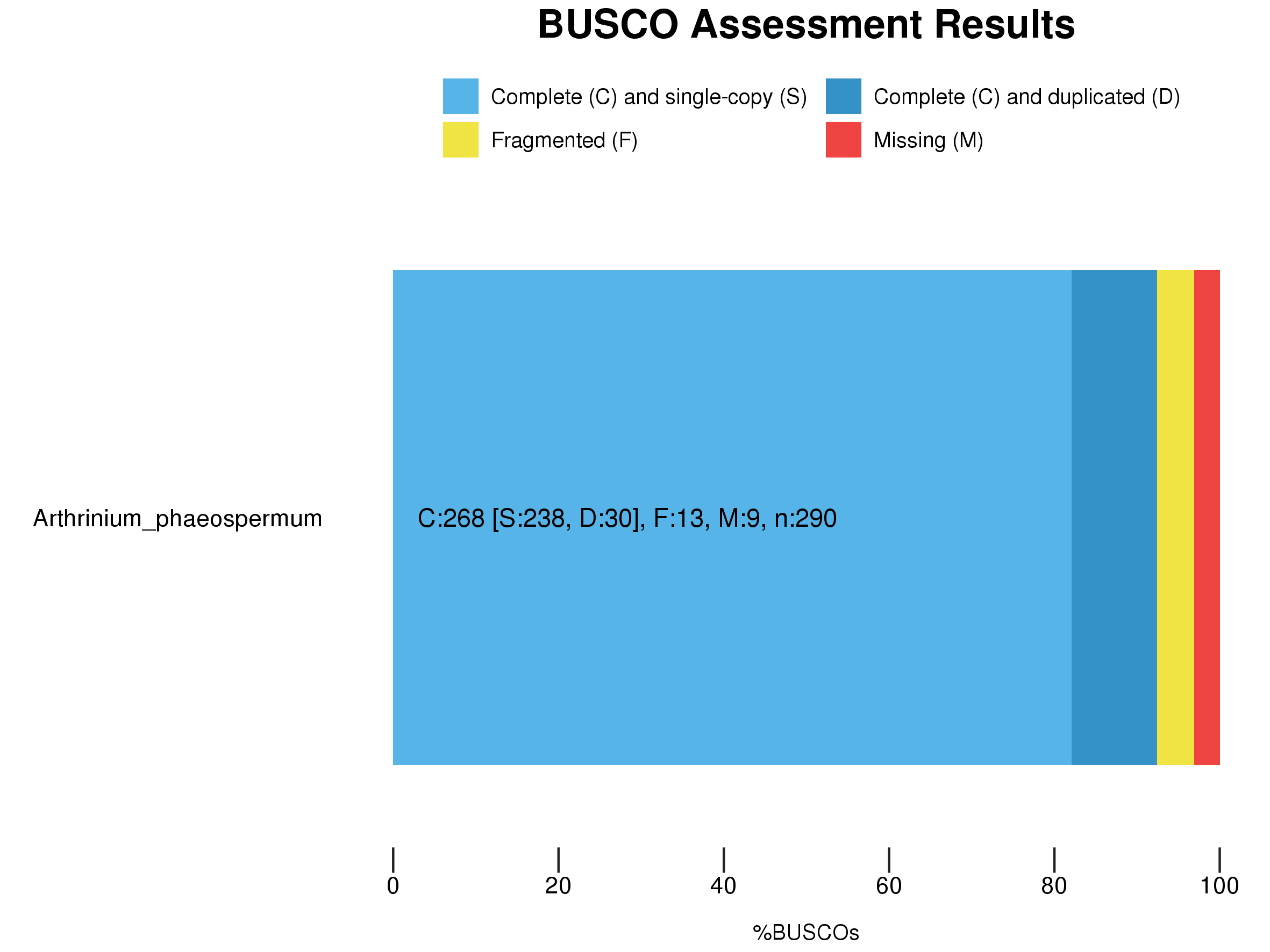

Supplement: Supplementary file 1 [file jof-07-01001-s001.zip › Figure S2. Test results of sequence assembly quality.jpg]

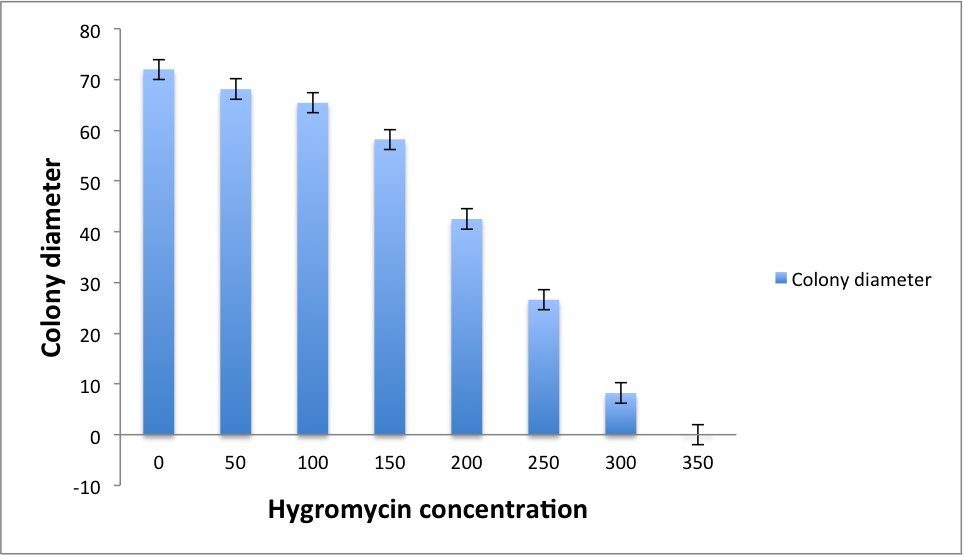

Supplement: Supplementary file 1 [file jof-07-01001-s001.zip › Figure S3 The kill curve for hygromycin.png]

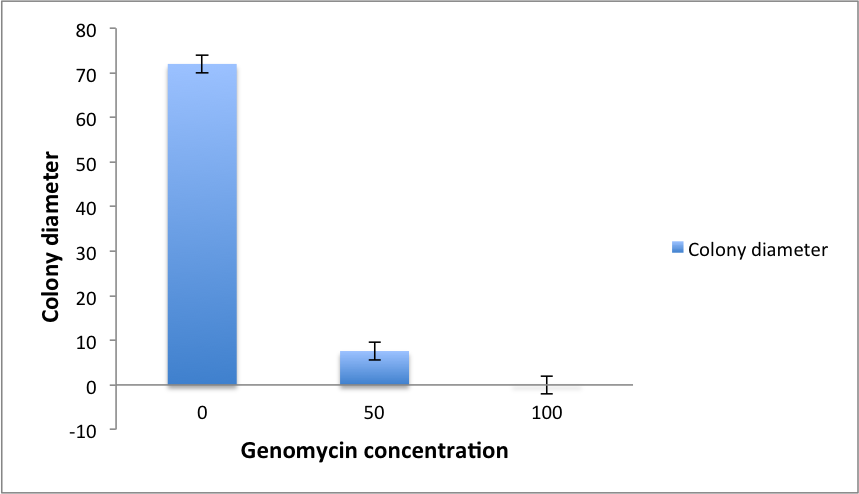

Supplement: Supplementary file 1 [file jof-07-01001-s001.zip › Figure S4The kill curve for genomycin.png]

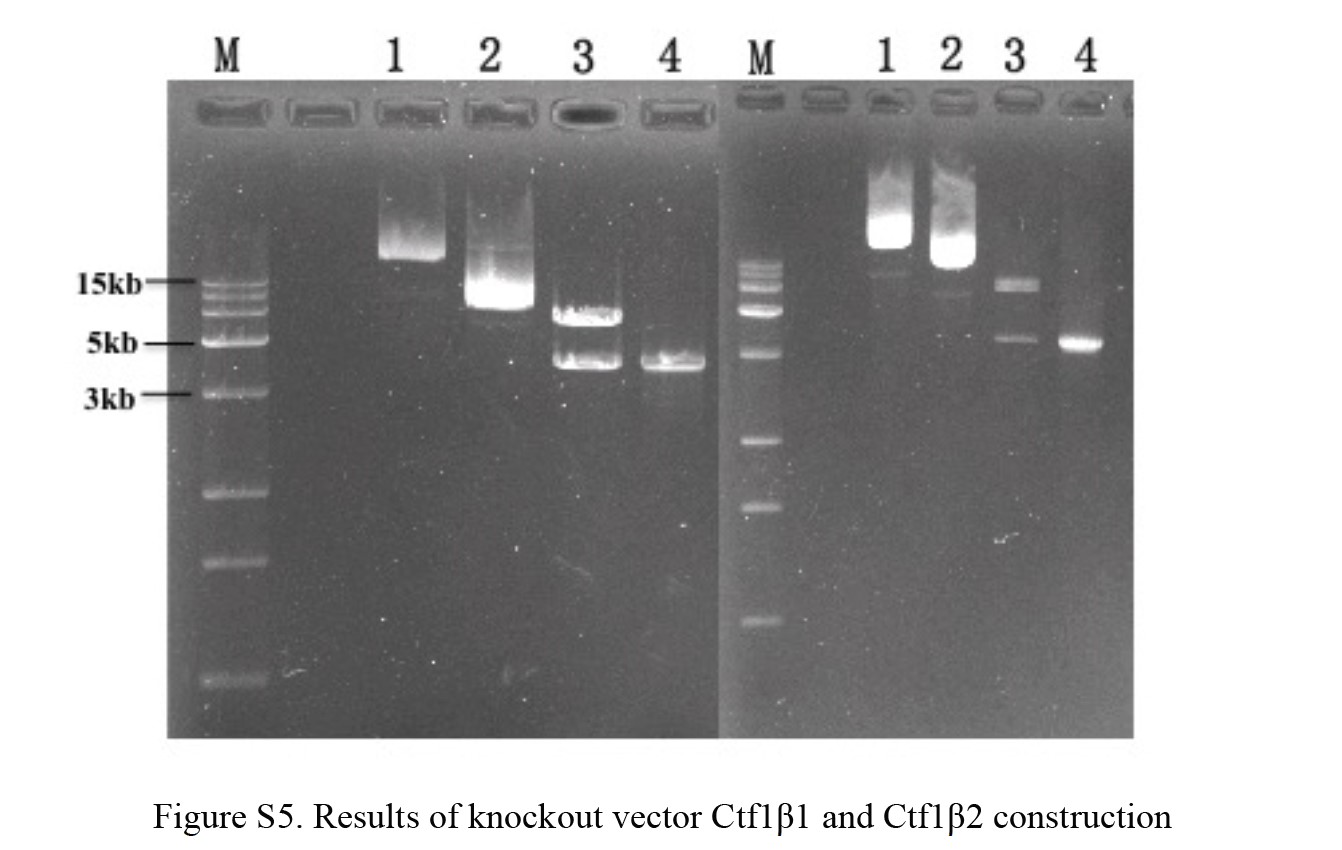

Supplement: Supplementary file 1 [file jof-07-01001-s001.zip › Figure S5. Results of knockout vector Ctf1a┬1 and Ctf1a┬2 construction.jpg]

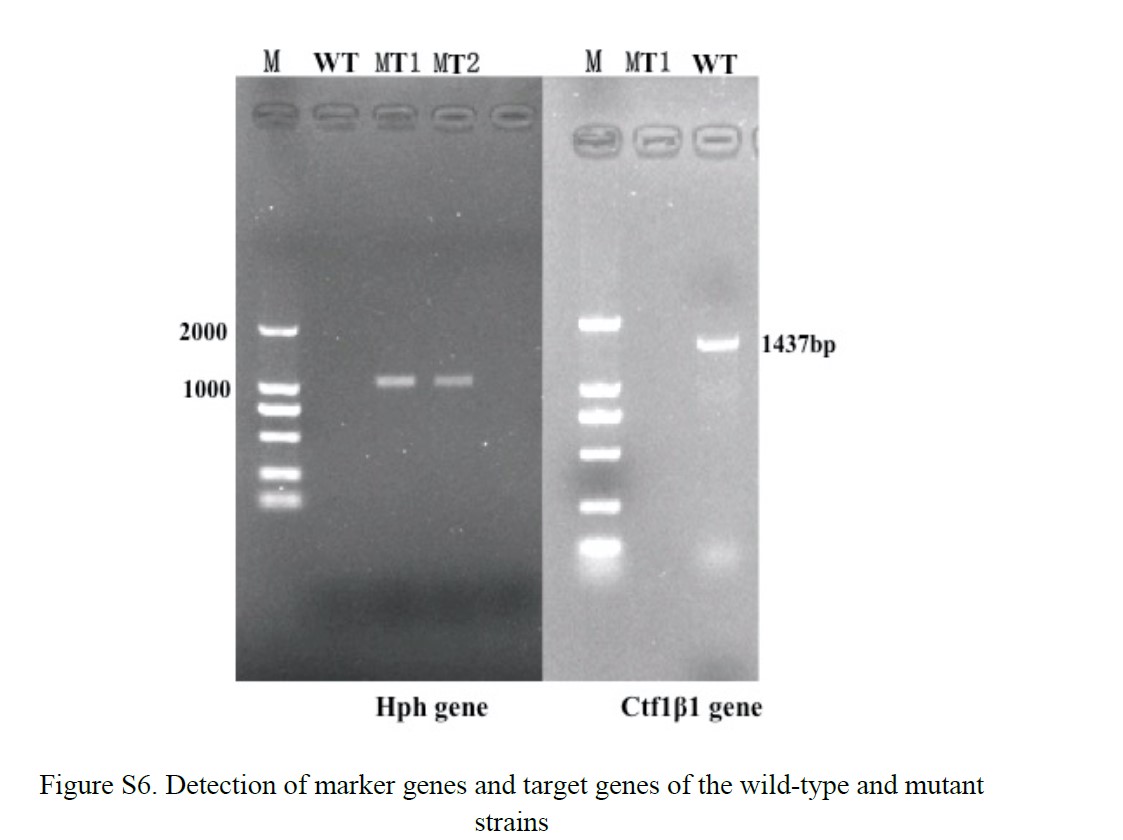

Supplement: Supplementary file 1 [file jof-07-01001-s001.zip › Figure S6.Detection of marker genes and target genes of the wild-type and mutant strains.jpg]

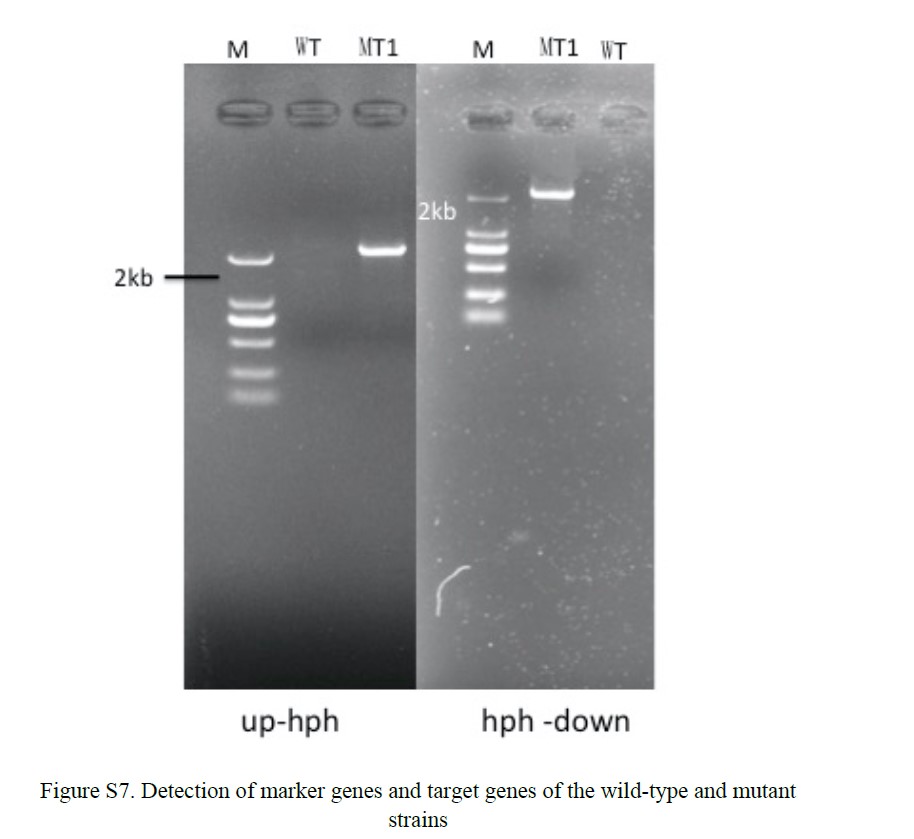

Supplement: Supplementary file 1 [file jof-07-01001-s001.zip › Figure S7. Detection of marker genes and target genes of the wild-type and mutant strains.jpg]

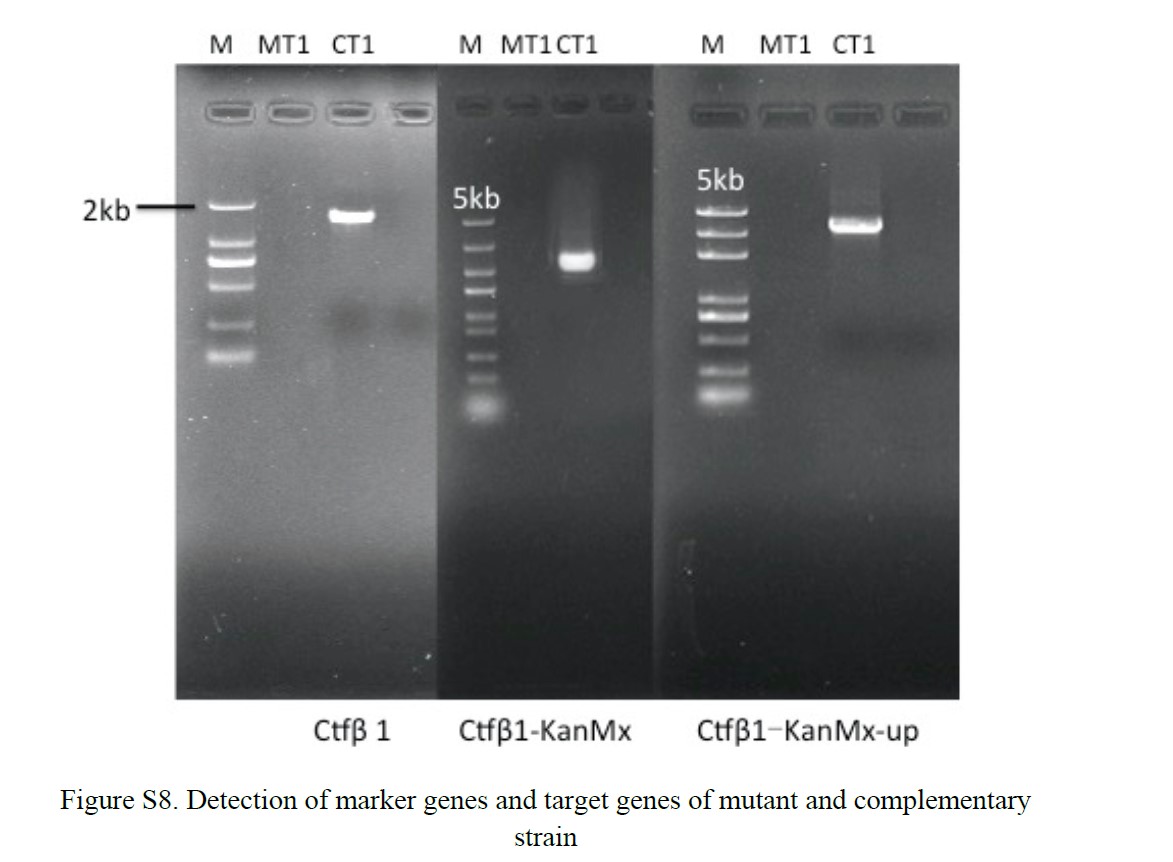

Supplement: Supplementary file 1 [file jof-07-01001-s001.zip › Figure S8. Detection of marker genes and target genes of mutant and complementary strains.jpg]
